# Steroidogenic pathway in girls diagnosed with autism spectrum disorders

**DOI:** 10.1371/journal.pone.0312933

**Published:** 2024-12-05

**Authors:** Katarina Jansakova, Martin Hill, Hana Celusakova, Gabriela Repiska, Marie Bicikova, Ludmila Macova, Katarína Polonyiova, Mária Kopcikova, Daniela Ostatnikova

**Affiliations:** 1 Institute of Physiology, Faculty of Medicine, Comenius University in Bratislava, Bratislava, Slovak Republic; 2 Department of Steroid Hormones and Proteohormones, Institute of Endocrinology, Prague, Czech Republic; University of Iceland, ICELAND

## Abstract

The diagnostic prevalence of autism spectrum disorders (ASD) shows boys to be more affected than girls. Due to this reason, there is a lack of research including and observing ASD girls. Present study was aimed to detect hormones of steroidogenesis pathway in prepubertal girls (n = 16) diagnosed with ASD and sex and age matched neurotypical controls (CTRL, n = 16). Collected plasma served for detection of conjugated and unconjugated steroids using gas chromatography tandem-mass spectrometry. We observed higher levels of steroids modulating ionotropic receptors, especially, GABAergic steroids and pregnenolone sulfate in ASD group. Concentration of many steroids throughout the pathway tend to be higher in ASD girls compared to CTRL. Pregnenolone and its isomers together with polar progestins and androstanes, i.e. sulfated steroids, were found to be higher in ASD group in comparison with CTRL group. Based on steroid product to precursor ratios, ASD group showed higher levels of sulfated/conjugated steroids suggesting higher sulfotransferase or lower steroid sulfatase activity and we also obtained data indicating lower activity of steroid 11β-hydroxylase compared to CTRL group despite higher corticosterone level observed in ASD. These findings need to be generalized in future studies to examine both genders and other age groups.

## Introduction

Autism spectrum disorders (ASD) cover a group of neurodevelopmental disorders with a still unknown etiology. Current ratio of males and females diagnosed with ASD is approximately 4:1 depending on the severity and module of ASD but some studies propose that the true male-to-female ratio is rather closer to 3:1 [1, 2]. This sex bias may be caused by several biological or psychological aspects like already described camouflaging of female individuals, non-adapted diagnostical test for females or it is questionable if females are truly less affected by ASD and/or more protected from ASD development [3–5].

As noted, ASD represents neurodevelopmental disorder, so its formation originates in prenatal period. During pregnancy, maternal organism adapts to a new physiological situation accompanying by hormonal changes necessary for fetal development and maternal-fetal communication/interaction via placenta [6, 7]. From the very first moments of fertilization to delivery, hormones are implicated in all processes responsible for successful implantation, adaptation of immune system to growing embryo/fetus, meeting the nutritional demands and, importantly, development of individual organs [8, 9] including brain [10].

Presented bias still attracts an attention toward sex hormones and their effect during prenatal development and also toward a trace left by this prenatal effect left during postnatal development [11]. Androgens can be formed via two ways and so classical front-door pathway and an alternative backdoor pathway [12]. The classical frontdoor pathway utilizes a precursor pregnenolone which is further converted to dehydroepiandrosterone (DHEA), subsequently to androstenedione and then to testosterone (TST) and dihydrotestosterone (DHT). On the other hand, androgens created via alternative backdoor pathway use 17-OH progesterone, which is further converted to 17-OH dihydroprogesterone and then to androsterone and androstanediol, bypassing common intermediates such as androstenedione and TST.

Frontdoor pathway, and its implication during fetal development, especially sex development, has been widely described but backdoor pathway was found to be as important as well [13]. Moreover, hormones involved in the classical frontdoor pathway of androgen production are mostly discussed in relation to ASD risk and ASD-related health conditions like polycystic ovary syndrome (PCOS) [14, 15]. Hormones involved in the backdoor pathway may be involved in ASD etiopathogenesis as well as it is expected from hormones of the frontdoor pathway [11].

The alternative backdoor pathway has not yet been described very often in individuals with ASD, but may be related to other pathological conditions mentioned in the context of ASD. This pathway participates in fetus masculinization and its activity is presented in placenta as well [13, 16]. In addition, higher expression of genes involved in this pathway was observed in women with PCOS, a medical condition that has been associated with the development of ASD in further generation [17].

Since androgens are dominantly male sex hormones, they do not primarily evoke they association with females. Fortunately, research is beginning to pay more attention to female individuals with ASD, but more studies are needed in this field [18, 19]. The aim of this study was to assess the steroidogenic pathway cascade in prepubertal girls diagnosed with ASD and in sex- and age-matched neurotypical controls. Moreover, steroid product to precursor ratios (PPRs) were investigated.

## Material and methods

The present study was approved by the Ethics Committee of the Faculty of Medicine of Comenius University and the University Hospital in Bratislava, Slovakia, in accordance with the 1964 Declaration of Helsinki and its subsequent amendments. The parents of all children enrolled in this study were informed of the study design and the written informed consent form was signed by both parents or caregivers of the respective child.

### Diagnosis of autism

Children evaluated by pediatrician or psychologist suspected for the presence of ASD were recruited and diagnosed in Academic research center for autism, Comenius University Faculty of Medicine in Bratislava. Children were diagnosed using diagnostic tools Autism Diagnostic Observation Schedule 2^nd^ revision (ADOS-2) [20] and Autism Diagnostic Interview-Revised (ADI-R) [21] and in line with Diagnostic and Statistical Manual of Mental Disorders 5^th^ edition (DSM-5) [22].

ADOS-2 is based on the behavioral observation of ASD symptoms. It is suitable for children of age from 12 months as well as for adults and it is applicable for individuals on different developmental levels and language skills. It comprises of various situations of structured and non-structured character in which social, communicative and other behaviors, relevant for ASD diagnosis are observed and evaluated accordingly. ADOS-2 consists of 5 diagnostic modules, selected after considering the age of a participant and his/her expressive language quality. Module 1 includes individuals older than 30 months, with speech limited to using a few words, without the use of complex phrases or whole sentences. Module 2 is intended for children who communicate in simple phrases and sentences. Module 3 was chosen for individuals using fluent, grammatically correct speech. The module 1 consists of 10 tasks, module 2 and 3 both include 14 tasks, with some of the tasks in consecutive modules overlapping. During these structured tasks a trained administrator examines social interaction and communication abilities of a participant. Immediately after finishing, the administrator assigns scores to the observed behaviors following the diagnostic algorithm. Afterwards, overall ADOS-2 score is calculated as well as scores for two separate domains: Social affect and Restricted and repetitive behaviors. In order to allow comparing the results of children from different modules, a calibrated score has been introduced.

ADI-R investigates ASD symptoms from other point of view and, thus, it is an interview with the parent or a caregiver of a suspected child. It looks for a developmental history of the individual together with the presence of the ASD-related most significant behavioral patterns. This diagnostic tool provides categorical results for three subdomains as follow: quality of social interaction (A); communication and language (B); and repetitive, restricted and stereotyped interests and behavior (C).

ASD diagnostic assessment was performed by a trained psychologist. The diagnosis was set after consensus of both diagnostic procedures together with a clinical judgement of a corresponding child. Children who did not meet described criteria despite their social and communication impairment were not included into the study. Other exclusion criteria included the presence of a systemic/acute disease, other disorder than ASD and using steroidal or non-steroidal medication treatment.

### Participants and sample collection

A total number of 32 girls, sixteen of them were girls diagnosed with ASD 4.69±1.16 years (shown as mean ± SD), and 16 were age and sex matched neurotypical controls (CTRL) 4.0±1.11 years. In the research ASD group, 11 children were diagnosed in module 1, 3 were diagnosed in module 2, and, 2 in module 3. Participants in CTRL group represented individuals with no previous history of ASD or other neurodevelopmental disorder and were chose by a pediatrician.

Blood was collected during morning hours after ADOS-2 diagnostic procedure.

### Analysis of steroid hormones

All collected plasma samples were used for detection of steroids together with their polar conjugates using gas chromatography tandem-mass spectrometry (GC-MS/MS). The analysis was performed according to Hill et al. [23].

### Statistical analysis

Whole analysis was performed as stated previously [24]. Age-adjusted ANCOVA (Statgraphics centurion XV, Statpoint, Inc., Herndon, Virginia, USA) was applied for the separation/distinguishing between two dependent variables represented by age and group represented by health status, CTRL and ASD (p<0.05 was considered as statistically significant). Homoscedasticity and data symmetry were achieved by power transformation before further data processing. Data are presented as re-transformed means with their lower and upper 95% confidence intervals (CI). For the statistical power of the factor Status (patients vs. controls) = 0.8 at statistical significance p = 0.05 and 16 participants in each group relative to the correlation coefficient with the covariate Age R = (0, 0.9), the estimated effect size η_p_^2^ ranged (0.223, 0.512) while the η_p_^2^ for individual steroids that differed significantly between patients and controls (p<0.05) was 0.301 (0.149, 0.715) (shown as median with range) and the η_p_^2^ for individual steroid PPRs that differed significantly between patients and controls (p<0.05) was 0.333 (0.14, 0.737). Thus, the power of the Status factor in the linear model for key steroids and PPRs was sufficient. The power analysis was completed using a statistical software PASS 2023 (Power Analysis and Sample Size Software (2023)) from NCSS, LLC. (Kaysville, Utah, USA).

## Results

A total number of 64 endogenous conjugated and unconjugated steroids across the steroidogenic pathway, were analyzed by GC-MS/MS in plasma of prepubertal girls diagnosed with ASD referred to as the ASD group and neurotypical matched controls, the CTRL group. The concentrations of all detected steroids are presented as mean values with their 95% upper/lower confidence intervals in Table 1.

**Table 1:** Levels (nM) of all detected steroids.

Steroid	ASD	CTRL	F-statistic	p-value
**Pregnenolone [nM]**	**0.902 (0.763, 1.07)**	**1.32 (1.12, 1.56)**	**5.16**	**0.032**
**Pregnenolone sulfate [nM]**	**45.7 (38, 54.9)**	**28.8 (23.8, 34.7)**	**6.2**	**0.019**
20α-Dihydropregnenolone [nM]	1 (0.864, 1.17)	1.05 (0.89, 1.23)	0.07	0.799
**20α-Dihydropregnenolone sulfate [nM]**	**235 (202, 273)**	**156 (132, 183)**	**6.96**	**0.015**
**17-Hydroxypregnenolone [nM]**	**3.26 (2.55, 4.17)**	**1.39 (1.07, 1.79)**	**11.62**	**0.002**
**17-Hydroxypregnenolone sulfate [nM]**	**4.41 (3.63, 5.35)**	**2.58 (2.08, 3.18)**	**7.06**	**0.013**
**16α-Hydroxypregnenolone [pM]**	**74.5 (57.5, 94.5)**	**38 (27.1, 51.3)**	**5.83**	**0.023**
Dehydroepiandrosterone (DHEA) [pM]	642 (536, 776)	682 (548, 859)	0.09	0.77
**DHEA sulfate [nM]**	**101 (71.3, 142)**	**34.5 (23.2, 50.9)**	**8.52**	**0.007**
7β-Hydroxy-DHEA [pM]	201 (179, 228)	208 (185, 238)	0.09	0.76
**Androstenediol [pM]**	**55.3 (38.5, 77)**	**13 (7.21, 21.8)**	**11.12**	**0.003**
Androstenediol sulfate [nM]	10.1 (7.15, 14.2)	16.9 (11.5, 24.6)	2.01	0.168
**5-Androstene-3β,16α,17β-triol [pM]**	**54.4 (38.9, 74.2)**	**20.7 (13.2, 31.1)**	**6.84**	**0.014**
5-Androstene-3β,16α,17β-triol sulfate [nM]	10.6 (7.33, 15.8)	5.72 (4.19, 8.03)	3.09	0.09
20α-Dihydroprogesterone [pM]	36.3 (26.6, 50.3)	41.3 (29.8, 58.4)	0.16	0.691
Conjugated 20α-dihydroprogesterone [pM]	424 (372, 474)	433 (377, 488)	0.03	0.854
17-Hydroxyprogesterone [pM]	318 (248, 425)	290 (224, 390)	0.12	0.734
**16α-Hydroxyprogesterone [pM]**	**102 (81.2, 131)**	**182 (137, 248)**	**4.81**	**0.038**
**17,20α-Dihydroxy-pregnene-3-one [pM]**	**119 (98.7, 143)**	**75.5 (62.8, 91.3)**	**6.05**	**0.022**
**Conjugated 17,20α-dihydroxy-pregnene-3-one [nM]**	**2.21 (1.65, 2.89)**	**1.09 (0.753, 1.52)**	**5.07**	**0.033**
Androstenedione [pM]	236 (167, 326)	119 (79.3, 173)	3.69	0.065
Conjugated testosterone [nM]	1.76 (1.08, 2.9)	1.18 (0.733, 1.91)	0.69	0.415
Epitestosterone sulfate [pM]	142 (92.9, 216)	173 (114, 263)	0.22	0.64
Estrone sulfate [pM]	103 (70.3, 147)	208 (147, 287)	4.1	0.053
**Allopregnanolone [pM]**	**43.7 (35.5, 52.6)**	**17 (12, 22.7)**	**15.1**	**<0.001**
**Allopregnanolone sulfate [nM]**	**2.05 (1.74, 2.4)**	**0.727 (0.608, 0.868)**	**37.73**	**<0.001**
Isopregnanolone [pM]	83.4 (65.2, 106)	116 (91.2, 145)	1.89	0.18
**Isopregnanolone sulfate [nM]**	**4.46 (3.86, 5.12)**	**3.17 (2.68, 3.72)**	**5.1**	**0.032**
**Pregnanolone sulfate [nM]**	**3.26 (2.67, 3.96)**	**1.69 (1.35, 2.11)**	**9.85**	**0.004**
**Epipregnanolone sulfate [nM]**	**1.07 (0.969, 1.17)**	**0.545 (0.451, 0.64)**	**29.34**	**<0.001**
17-Hydroxyallopregnanolone sulfate [nM]	1.5 (1.27, 1.77)	1.2 (0.999, 1.42)	1.84	0.187
Conjugated 17-hydroxypregnanolone [nM]	1.6 (1.35, 1.87)	1.62 (1.37, 1.9)	0.01	0.911
5α,20α-Tetrahydroprogesterone [pM]	65.7 (55.6, 76.2)	57.5 (47.4, 68.1)	0.63	0.435
Conjugated 5α,20α-tetrahydroprogesterone [pM]	89.2 (65.1, 119)	138 (104, 180)	2.38	0.135
5α-Pregnane-3α,20α-diol [pM]	258 (185, 347)	236 (165, 323)	0.08	0.779
Conjugated 5α-pregnane-3α,20α-diol [nM]	19.8 (15.2, 25.2)	12.3 (9.13, 16.2)	3.21	0.085
5α-Pregnane-3β,20α-diol [pM]	197 (134, 288)	348 (239, 504)	2.23	0.147
Conjugated 5α-pregnane-3β,20α-diol [nM]	139 (118, 164)	107 (91.4, 126)	2.54	0.125
5β,20α-Tetrahydroprogesterone [pM]	22.6 (18.4, 28.7)	22.9 (18.6, 29.2)	0	0.947
Conjugated 5β,20α-tetrahydroprogesterone [pM]	59.7 (47.4, 76.4)	54.4 (43.2, 69.7)	0.15	0.698
Conjugated 5β-pregnane-3α,20α-diol [nM]	2.68 (2.12, 3.41)	2.08 (1.65, 2.64)	1.14	0.295
Conjugated 5β-pregnane-3β,20α-diol [nM]	15.1 (10.8, 20.9)	12.4 (8.71, 17.5)	0.34	0.566
**5α-Pregnane-3α,17,20α-triol [pM]**	**17.8 (10, 30.4)**	**1.93 (0.849, 4.03)**	**11.55**	**0.002**
**Conjugated 5α-pregnane-3α,17,20α-triol [nM]**	**127 (86.6, 205)**	**20.5 (17.4, 24.4)**	**52.67**	**<0.001**
**5β-Pregnane-3α,17,20α-triol [pM]**	**315 (246, 415)**	**172 (138, 218)**	**6.18**	**0.02**
**Conjugated 5β-pregnane-3α,17,20α-triol [nM]**	**35.3 (28.6, 43.6)**	**14.7 (11.9, 18.1)**	**18.44**	**<0.001**
5α-Androstane-3,17-dione [pM]	21.8 (15.8, 28.9)	20.1 (14.2, 27.2)	0.07	0.799
**Androsterone [pM]**	**91 (72.2, 113)**	**29.5 (20.2, 41.3)**	**16.28**	**<0.001**
**Androsterone sulfate [nM]**	**76.4 (59.3, 113)**	**18.7 (14, 30)**	**21.16**	**<0.001**
Epiandrosterone [pM]	24.3 (18.6, 37.1)	25 (19, 39.2)	0.01	0.921
**Epiandrosterone sulfate [nM]**	**19.3 (15.3, 27.3)**	**6.55 (5.05, 9.88)**	**14.91**	**<0.001**
Etiocholanolone [pM]	29.3 (22.9, 42.5)	34 (26.8, 48.4)	0.3	0.589
**Etiocholanolone sulfate [nM]**	**1.68 (1.45, 2.02)**	**0.746 (0.623, 0.949)**	**20.93**	**<0.001**
**Conjugated 5α-androstane-3α,17β-diol [nM]**	**1.09 (0.898, 1.42)**	**0.369 (0.298, 0.502)**	**22.64**	**<0.001**
Conjugated 5α-androstane-3β,17β-diol [nM]	1.49 (1.14, 2.32)	0.797 (0.594, 1.32)	3.71	0.066
**Conjugated 5β-androstane-3α,17β-diol [pM]**	**118 (102, 143)**	**214 (183, 261)**	**13.61**	**0.001**
Cortisol [nM]	215 (184, 248)	243 (209, 279)	0.69	0.412
**Corticosterone [nM]**	**6.43 (5.22, 8.05)**	**2.95 (2.48, 3.55)**	**16.44**	**<0.001**
11β-Hydroxyandrostenedione [nM]	15 (12.3, 18)	19.7 (16.7, 23)	2.47	0.127
**11β-Hydroxyandrosterone [nM]**	**1.1 (0.951, 1.28)**	**2.26 (1.9, 2.71)**	**20.12**	**<0.001**
**11β-Hydroxyandrosterone sulfate [nM]**	**6.18 (5.33, 7.25)**	**4.14 (3.62, 4.76)**	**7.9**	**0.01**
11β-Hydroxyepiandrosterone sulfate [nM]	0.719 (0.489, 1.06)	0.565 (0.387, 0.833)	0.39	0.539
**11β-Hydroxyetiocholanolone [nM]**	**0.547 (0.469, 0.641)**	**1.02 (0.871, 1.19)**	**15.58**	**<0.001**
**11β-Hydroxyetiocholanolone sulfate [nM]**	**30.6 (23.7, 39.8)**	**17.4 (13.2, 23)**	**4.56**	**0.042**

Steroid assessment in the groups of girls diagnosed with autism spectrum disorders (ASD) and corresponding controls (CTRL); shown as means with 95% confidence intervals (after age adjustment); significant differences are in bold

Looking at the beginning of the pathway, significantly lower concentration of pregnenolone (F = 5.2, p = 0.032) was observed in ASD group in comparison with CTRL group. On the other hand, several other C21 Δ^5^ steroids involved in the metabolism of pregnenolone like 20α-dihydropregnenolone sulfate (F = 7, p = 0.015), 17-hydroxypregnenolone and its sulfate (F = 11.6, p = 0.002 and F = 7.1, p = 0.013, respectively) or 16α-hydroxypregnenolone showed significantly higher concentration in ASD (F = 5.8, p = 0.023) but 16α-hydroxypregnenolone sulfate (F = 15.9, p<0.001) was significantly lower in ASD compared to CTRL.

Regarding the adrenal precursors of TST and their metabolites, some of them tended to higher values in ASD group. However, the TST itself was not possible to detect while its conjugate and sulfated epimer, epitestosterone sulfate did not significantly differ between analyzed groups (F = 0.7, p = 0.415 and F = 0.2, p = 0.64, respectively). The concentration of DHEA (the metabolite of 17-hydroxypregnenolone) was comparable between analyzed groups while its sulfate was significantly higher in ASD group (F = 0.1, p = 0.77 and F = 8.5, p = 0.007, respectively). Androstenediol, a metabolite of DHEA, showed significantly higher concentration in ASD group (F = 11.1, p = 0.003) while its conjugate did not significantly differ between groups (F = 2, p = 0.168). Androstenedione, a metabolite of DHEA, showed a borderline trend towards higher concentration in the ASD group compared to the CTRL group (F = 3.7, p = 0.065), while 17-OH progesterone did not differ between groups (F = 0.1, p = 0.734). Estrone sulfate, a sulfated metabolite of estrone formed from androstenedione, showed a borderline trend towards lower concentrations in ASD (F = 4.1, p = 0.053). The 5α/β-reduced 17-hydroxyprogesterone metabolites such as 5α-pregnane-3α,17,20α-triol (F = 11.6, p = 0.002) and its conjugate (F = 52.7, p<0.001) and 5β-pregnane-3α,17,20α-triol and its conjugate (F = 6.2, p = 0.02 and F = 18.4, p<0.001, respectively) were significantly higher in the ASD group compared to the CTRL group. Also, 17-deoxy-5α/β reduced pregnanes involved in the backdoor pathway of androgen formation, such as allopregnanolone (F = 15.1, p<0.001) and its sulfate (F = 37.7, p<0.001), isopregnanolone sulfate (F = 5.1, p = 0.032), pregnanolone sulfate (F = 9.9, p = 0.004) and epipregnanolone sulfate (F = 29.3, p<0.001) were significantly higher in ASD compared to CTRL and conjugated 5α-pregnane-3α,20α-diol (F = 3.2, p = 0.085) also tended to be higher in the ASD group compared to CRTL. In addition, the 5α-reduced C19 steroids androsterone and its sulfate (F = 16.3, p<0.001 and F = 21.2, p<0.001, respectively) and conjugated 5α-androstane-3α,17β-diol (F = 22.6, p<0.001) were elevated in ASD, and there was also a suggestion of an increase in conjugated 5α-androstane-3β,17β-diol (F = 3.7, p = 0.066). Although epiandrosterone and etiocholanolone did not show significant differences between the study groups (F = 0, p = 0.921 and F = 0.3, p = 0.589, respectively), their sulfates were also significantly higher in the ASD group (F = 14.9, p<0.001 and F = 20.9, p<0.001, respectively).

Evaluation of the PPRs offers to look at the activity of hormone-converting enzymes (Table 2). The ratio of conjugated to unconjugated steroids is indicative of SULT2A1 transferase activity, where significantly higher values were observed in ASD for pregnenolone (F = 35.3, p<0.001) and its metabolites 20α-hydroxypregnenolone (F = 5, p = 0.034) and 16α-hydroxypregnenolone (F = 6.8, p = 0.016). Furthermore, some PPRs reflecting the balance between conjugated and free 5α/β-pregnanes showed significantly higher ratio in ASD compared to CTRL such as isopregnanolone (F = 8.7, p = 0.006), 5α-pregnan-3β,20α-diol (F = 6, p = 0.002). The same trend was also observed for 5α/β-androstanes epiandrosterone (F = 47.8, p<0.001), 11β-hydroxyandrosterone (F = 38.6, p<0.001) and 11β-hydroxyetiocholanolone (F = 56.4, p<0.001). The second and third of these steroids may originate from the cleavage of cortisol by CYP17A1 and/or from the 11β-hydroxylation of 11-deoxy-androstanes [25]. Interestingly, a significantly lower ratio of sulfated androstenediol to its unconjugated counterpart was observed in the ASD group (F = 10.7, p = 0.003), whereas this ratio did not differ significantly between groups for DHEA (F = 2.8, p = 0.107).

**Table 2:** Product to precursor ratios reflecting the activities of steroidogenic enzymes.

Steroid ratios reflecting the activities of steroidogenic enzymes	ASD	CTRL	F-statistic	p-value
**Sulfotransferase (SULT2A1) vs. sulfatase (STS)**				
**Pregnenolone, C/U**	**46.1 (38.5, 55.8)**	**18.7 (16.5, 21.3)**	**35.3**	**<0.001**
**20α-Dihydropregnenolone, C/U**	**206 (170, 250)**	**133 (109, 162)**	**5**	**0.034**
17-Hydroxypregnenolone, C/U	1.45 (1.27, 1.64)	1.56 (1.38, 1.76)	0.4	0.537
Dehydroepiandrosterone (DHEA), C/U	112 (83.4, 143)	67.5 (46.4, 92.2)	2.8	0.107
**Androstenediol, C/U**	**146 (92.6, 240)**	**933 (487, 1950)**	**10.7**	**0.003**
5-Androstene-3β,16α,17β-triol, C/U	243 (168, 357)	405 (275, 604)	1.7	0.201
20α-Dihydroprogesterone, C/U	10 (7.45, 13.5)	10.4 (7.68, 14)	0	0.917
Allopregnanolone (3α,5α-THP), C/U	45.9 (38.2, 57)	47.5 (39.3, 59.3)	0	0.867
**Isopregnanolone (3β,5α-THP), C/U**	**47.7 (37.4, 61.9)**	**24.4 (20, 30.2)**	**8.7**	**0.006**
5α,20α-Tetrahydroprogesterone, C/U	1.62 (1.14, 2.37)	2.3 (1.64, 3.31)	1	0.333
5α-Pregnane-3α,20α-diol, C/U	67 (45.1, 107)	50.8 (35.9, 75.8)	0.5	0.49
**5α-Pregnane-3β,20α-diol, C/U**	**547 (399, 746)**	**248 (176, 347)**	**6**	**0.022**
**10** ^**−3**^ **·5α-Pregnane-3α,17,20α-triol, C/U**	**2.87 (1.53, 5.63)**	**36.4 (16.5, 85.9)**	**12.5**	**0.002**
5β,20α-Tetrahydroprogesterone, C/U	2.47 (1.78, 3.32)	2.42 (1.7, 3.34)	0	0.955
5β-Pregnane-3α,17,20α-triol, C/U	69.4 (54.9, 87.6)	85 (66.8, 108)	0.8	0.39
Androsterone (3α,5α-THA), C/U	742 (474, 1190)	858 (544, 1380)	0.1	0.756
**Epiandrosterone (3β,5α-THA), C/U**	**1050 (848, 1290)**	**177 (126, 244)**	**47.8**	**<0.001**
Etiocholanolone (3α,5β-THA), C/U	66.9 (52.6, 83.2)	50.7 (39, 64.1)	1.4	0.255
**11β-Hydroxyandrosterone, C/U**	**4.63 (4.05, 5.37)**	**2.29 (2.1, 2.51)**	**38.6**	**<0.001**
**11β-Hydroxyetiocholanolone, C/U**	**100 (80, 125)**	**15.7 (11.8, 20.7)**	**56.4**	**<0.001**
**C17-Hydroxylase-C17,20-lyase (CYP17A1)–hydroxylase step**				
**17-Hydroxypregnenolone/pregnenolone**	**3.33 (2.8, 3.98)**	**1.07 (0.898, 1.28)**	**41.9**	**<0.001**
10^3^·17-Hydroxypregnenolone/pregnenolone, sulfates	119 (102, 138)	88.9 (75.4, 104)	3.5	0.071
17,20α-dihydroxy-4-pregnen-3-one/20α-dihydroprogesterone	3.15 (2.41, 4.15)	2.23 (1.66, 3.03)	1.5	0.231
**17,20α-dihydroxy-4-pregnen-3-one/20α-dihydroprogesterone, conjugates**	**5.25 (4.36, 6.21)**	**2.24 (1.61, 2.95)**	**14.3**	**0.001**
**Cortisol/corticosterone**	**34.1 (28.5, 40.9)**	**50.8 (41.5, 62.6)**	**4.5**	**0.046**
**17-Hydroxyallopregnanolone/allopregnanolone, sulfates**	**0.789 (0.728, 0.857)**	**1.65 (1.48, 1.86)**	**61.8**	**<0.001**
**17-Hydroxypregnanolone/pregnanolone, conjugates**	**0.455 (0.388, 0.526)**	**1.12 (1.02, 1.23)**	**61.9**	**<0.001**
10^3^·5α-Pregnane-3α,17,20α-triol/5α-pregnane-3α,20α-diol	**63 (30.7, 137)**	**14.1 (7.42, 28.1)**	**4.5**	**0.042**
5α-Pregnane-3α,17,20α-triol/5α-pregnane-3α,20α-diol, conjugates	4.04 (2.32, 7.62)	3.06 (1.82, 5.54)	0.2	0.633
**5β-Pregnane-3α,17,20α-triol/5β-pregnane-3α,20α-diol, conjugates**	**12.9 (11.3, 15)**	**8.93 (8.01, 10)**	**9.5**	**0.007**
**C17-Hydroxylase-C17,20-lyase (CYP17A1)—lyase step**				
**10** ^**3**^ **·DHEA/17-hydroxypregnenolone**	**240 (204, 275)**	**329 (292, 364)**	**6.3**	**0.019**
DHEA/17-hydroxypregnenolone, sulfates	22.9 (17.8, 29.6)	13.3 (9.9, 17.8)	4	0.058
10^3^·Androstenedione/17-hydroxyprogesterone	568 (461, 705)	425 (349, 521)	2	0.169
10^3^·11β-Hydroxyandrostenedione/cortisol	71 (66.4, 76)	71.5 (66.4, 77.2)	0	0.92
Androsterone/17-hydroxyallopregnanolone, sulfates	39.6 (28.2, 56.1)	19.9 (14.1, 28.3)	4	0.057
Androsterone/5α-pregnane-3α,17,20α-triol	5.02 (2.87, 8.76)	11.6 (6.39, 20.9)	2.2	0.155
Androsterone/5α-pregnane-3α,17,20α-triol, conjugates	1.01 (0.665, 1.55)	0.772 (0.509, 1.2)	0.4	0.536
10^3^·Etiocholanolone/5β-pregnane-3α,17,20α-triol	94.5 (75.8, 119)	152 (118, 198)	3.9	0.06
10^3^·Etiocholanolone/5β-pregnane-3α,17,20α-triol, conjugates	63.6 (52.6, 78.5)	78.7 (64.2, 99)	1.1	0.302
**11β-Hydroxylase (CYP11B1)**				
**11β-Hydroxyandrostenedione/androstenedione**	**58.2 (49, 70.2)**	**175 (132, 242)**	**23.6**	**<0.001**
**11β-Hydroxyandrosterone/androsterone**	**13.1 (9.84, 17.7)**	**69.5 (48, 102)**	**25.4**	**<0.001**
10^3^·11β-Hydroxyandrosterone/androsterone, sulfates	94.8 (68.2, 131)	153 (113, 207)	2.3	0.138
10^3^·11β-Hydroxyepiandrosterone/epiandrosterone, sulfates	54.1 (37.5, 75.2)	62.1 (43.3, 86.2)	0.2	0.692
**11β-hydroxyetiocholanolone/etiocholanolone**	**14.2 (12.6, 16.3)**	**36.8 (30.8, 44.5)**	**40.3**	**<0.001**
11β-hydroxyetiocholanolone/etiocholanolone, sulfates	16.8 (13.7, 20.2)	13.8 (11.1, 16.8)	1	0.33
**Aromatase (CYP19A1)**				
**Estrone sulfate/androstenedione**	**0.393 (0.236, 0.671)**	**1.51 (0.855, 2.74)**	**6**	**0.021**
**5β-Reductase (AKR1D1)**				
17-Hydroxypregnanolone sulfate/17-hydroxyprogesterone	3.31 (2.5, 4.33)	5.53 (4.32, 7)	4.1	0.055
5β-Pregnane-3α,17,20α-triol/17,20α-dihydroxy-4-pregnen-3-one	3.5 (2.98, 4.07)	2.58 (2.16, 3.05)	3.5	0.074
10^3^·5β,20α-Tetrahydroprogesterone /20α-dihydroprogesterone	898 (625, 1310)	488 (352, 685)	3.1	0.09
**10** ^**3**^ **·Etiocholanolone /androstenedione**	**111 (87, 143)**	**272 (205, 367)**	**11.5**	**0.002**
**Etiocholanolone sulfate/androstenedione**	**7.28 (5.69, 9.54)**	**12.9 (9.67, 17.9)**	**4.3**	**0.048**
11β-Hydroxyetiocholanolone sulfate/11β-hydroxyandrostenedione	1.47 (1.08, 1.93)	1.15 (0.818, 1.56)	0.6	0.434
10^3^·11β-Hydroxyetiocholanolone/11β-hydroxyandrostenedione	**38 (30.9, 46.2)**	**62.1 (51.8, 73.6)**	**6.9**	**0.014**
**Aldoketoreductases (AKR1C2,4)—**reduction **vs. 17β-hydroxysteroid dehydrogenases (HSD17B2,6)**—oxidation + 3β isomerization				
**10** ^**3**^ **·Allopregnanolone/isopregnanolone**	**422 (338, 526)**	**199 (153, 258)**	**10**	**0.004**
**10** ^**3**^ **·Allopregnanolone/isopregnanolone, sulfates**	**508 (462, 558)**	**281 (256, 309)**	**40**	**<0.001**
Pregnanolone/epipregnanolone, conjugates	3.45 (3.14, 3.79)	3.03 (2.73, 3.36)	1.8	0.195
10^3^·5α-Pregnane-3α,20α-diol/5α-pregnane-3β,20α-diol, conjugates	152 (125, 182)	125 (102, 149)	1.2	0.293
10^3^·5β-Pregnane-3α,20α-diol/5β-pregnane-3β,20α-diol, conjugates	200 (158, 258)	200 (157, 260)	0	0.997
**Androsterone/epiandrosterone**	**3.13 (2.25, 4.29)**	**0.974 (0.648, 1.43)**	**10.9**	**0.003**
Androsterone/epiandrosterone, sulfates	4.05 (3.75, 4.4)	3.61 (3.38, 3.87)	2.5	0.126
10^3^·5α-Androstane-3α,17β-diol/5α-androstane-3β,17β-diol, conjugates	585 (482, 739)	489 (416, 591)	0.9	0.359
11β-Hydroxyandrosterone/11β-hydroxyepiandrosterone, conjugates	7.16 (5.33, 9.66)	10.7 (7.86, 14.7)	1.7	0.2
**Aldoketoreductase (AKR1C3)**—reduction **vs. 17β-hydroxysteroid dehydrogenase (HSD17B2)—**oxidation				
10^3^·Androstenediol/DHEA	76.1 (49.6, 115)	41.6 (24.9, 67.7)	1.7	0.2
**10** ^**3**^ **·Androstenediol/DHEA, sulfates**	**118 (80.1, 168)**	**319 (228, 437)**	**8.4**	**0.007**
**10** ^**3**^ **·5α-Androstane-3α,17β-diol/androsterone, conjugates**	**13.8 (13, 14.7)**	**18.9 (17, 21.6)**	**14.6**	**<0.001**
10^3^·5α-Androstane-3β,17β-diol/epiandrosterone, conjugates	121 (106, 139)	123 (108, 140)	0	0.906
**10** ^**3**^ **·5β-Androstane-3α,17β-diol/etiocholanolone, conjugates**	**58.9 (46.3, 76.3)**	**123 (87.4, 180)**	**6.3**	**0.019**

Analyses performed in the groups of girls diagnosed with autism spectrum disorders (ASD) and corresponding controls (CTRL); shown as means with 95% confidence intervals (after age adjustment); significant differences are in bold

The PPRs depicting CYP17A1 activity in the lyase step showed significantly lower ratio of DHEA to 17-hydroxypregnenolone (F = 6.3, p = 0.019).

The PPRs reflecting CYP17A1 activity in the hydroxylase step in Δ^5^ and Δ^4^ pathways were significantly higher in the ASD compared with the CTRL group, but these changes were not consistent in 5α/β-pregnanes (results summarized in Table 2).

Significantly reduced CYP11B1 activity in the ASD group was observed for the ratios of 11β-hydroxyetiocholanolone to etiocholanolone (F = 40.3, p<0.001) and 11β-androstenedione to androstenedione (F = 23. 6, p<0.001), 11β-hydroxyandrosterone to androsterone (F = 25.4, p<0.001), but this trend did not reach statistical significance for the ratio of conjugated 11β-hydroxyandrosterone to androsterone (F = 2.3, p = 0.138).

Considering the activity of AKR1C3, significantly lower PPRs were observed in ASD group in ratios androstenediol sulfate to DHEAS (F = 8.4, p = 0.007), conjugated 5α-androstan-3α,17β-diol to androsterone (F = 14.6, p<0.001), and conjugated 5β -androstan-3α,17β-diol to etiocholanolone.

Considering the activity of AKR1C2 and 4, higher PPRs were observed in ASD group in ratios allopregnanolone to isopregnanolone (F = 10, p = 0.004) and their conjugates (F = 40, p<0.001) as well as 5α-pregnan-3α, 17, 20α-triol to 5α-pregnan-3β, 17, 20α-triol (F = 9.6, p = 0.005) and androsterone to epiandrosterone (F = 10.9, p = 0.003).

## Discussion

To our knowledge, this is one of the few published studies looking at steroidogenesis in girls diagnosed with ASD and matched neurotypical controls. Since there is a lower number of diagnosed girls in comparison with male individuals, studies involving female individuals are underestimated and generally missing. This study aims to show the relationship between conjugated and unconjugated steroids and ASD vs. CTRL. Conjugated steroids generally include steroid sulfates and glucuronides. Steroid conjugates having a dominance of 3α/β monosulfates are called steroid sulfates while the term steroid conjugate is used for sulfates, disulfates and glucuronides [26]. Together 64 detected steroids help to create a picture of the whole steroidogenesis. Thus, the comparisons of obtained results with other studies are limited due to different methodology as well as samples used and/or distinct group of interest from ours regarding the age or ASD module.

In general, higher concentration of the majority of free and conjugated steroids were observed in ASD group compared to CTRL. These results are in line with the complex studies performed by Majewska et al. [27] and Gasser et al. [18]. Both these studies reported higher concentrations of steroids in ASD girls compared to CTRL ones detected in saliva and urine, respectively [18, 27]. These studies are the most suitable to compare our data with.

The formation of pregnenolone represents the first step in steroidogenesis and its levels were lower in patients with ASD, suggesting lower cholesterol desmolase (CYP11A1) activity (catalyzing pregnenolone synthesis) in these patients. Contrarywise, the higher levels of 17-hydroxy and 16α-hydroxypregnenolone in the ASD group compared to CTRL may be related to the higher activity of the 17-hydroxylase step of the C17-hydroxylase-C17,20 lyase (CYP17A1) catalyzing the conversion of free and sulfated pregnenolone [28] to the aforementioned metabolites in the adrenal *zona fasciculata*. In the past, relation of polymorphism in gene coding CYP17A1 and Asperger syndrome was observed [29]. Regarding the enzyme 11β-hydroxylase (CYP11B1), which is produced predominantly in the *zona fasciculata*, lower activity was observed in ASD individuals. Surprisingly, higher concentration of corticosterone was observed in ASD group. In this context, however, animal studies showed that corticosterone treatment led to abnormal emotional reactions and coping strategies [30, 31]. Many parents reported that girls tried to manage social difficulties by becoming more quiet or cautious in their communication [32]. Deficits in cognitive regulation of negative emotions are huge risk factor for mental health problems. There is a little number of studies monitoring the difference in emotional regulation in boys and girls with ASD. The specific case for ASD girls and women is menstrual cycle. They may feel pain, may suffer from increasing anxiety which could lead to misunderstanding feelings during cycle. Their sensory issues, repetitive behaviors and difficulties regulating emotions can be worse during period [33]. Despite having a strictly pre-pubertal, age-matched girls puberty onset seems to be a gradual process [24]. Treatment with cortisone can be helpful for autistic girls before and during their period due to the ability to support cognitive control of emotion regulation. It was found that overwhelming negative emotions were reduced within 30–90 minutes after cortisol treatment [34].

In contrast to lower pregnenolone, pregnenolone sulfate was higher in ASD group compared to CTRL group. Same result was observed by Majewska et al. in saliva of ASD pre-pubertal girls compared to controls [27]. Pregnenolone sulfate is a positive modulator of neuro-activating NMDA receptors, which hypofunction has been related to ASD and schizophrenia, while negative modulator of neuro-inhibiting GABA_A_R and glycine receptors [35]. Also, this steroid negatively modulates neuro-activating AMPA/KAR receptors, capsaicin TRPV1 receptors, associated with different types of neuro disorders including autism, which serve as a molecular gateway to the pain pathway and TRPC5 receptors, which participate in the pathophysiology of innate fear [36–38]. AMPA are Ca^2+^ permeable receptors and their mediated excitotoxicity was considered in the context of many neurological disorders e.g. schizophrenia, Alzheimer disease and others [39]. Mutations in AMPAR subunit GLuA2 have been related to ASD. Furthermore, the pregnenolone sulfate is a positive modulator of TRPM3 receptors, the Ca^2+^ permeable cation channels, which are involved in temperature and calcium homeostasis. Numerous novel variants of this gene have been found in patients with neurodevelopmental syndrome associated with TRPM3, who share some symptoms with ASD. De novo substitutions in TRPM3 gene were found to be associated with epilepsy, intellectual disability and developmental delay affecting motor and language skills [40, 41]. Deletion in this gene was also associated with autism phenotype [42]. The function of TRPC5 receptors, which are negatively modulated by pregnenolone sulfate as mentioned above, is importantly affected by calcium/calmodulin dependent kinase II β mediating the ability of this receptor to regulate neuronal morphogenesis. Possibly, the mutation in this gene and/or deregulation of this signaling could play a role in ASD development [43].

Besides, it seems that ASD group has higher concentration of GABAergic steroids. GABAergic synaptic dysregulation and related inhibitory and excitatory imbalance has been widely mentioned in relation to ASD [44]. It can be assumed that on the one hand, pregnenolone sulfate may play a protective role through compensatory mechanisms at various ionotropic receptors, but on the other hand, it may stimulate cognitive performance [37]. It stimulates NMDA receptors while suppress AMPA receptors and dysfunction of both these types of receptors contribute to ASD making pregnenolone sulfate as potential therapeutics.

In the urine, the major metabolite of progesterone 5β-pregnane-3α,20α-diol was higher in ASD girls compared to controls [45]. Other study performed by Gasser et al. [46] showed tendency to lower concentration of progesterone metabolites in autistic girls compared to controls. A few plasma markers measured in our study overlapped with this study, so 5β-pregnane-3α,20α-diol showed only a hint to lower levels in ASD girls compared to CTRL while 20α-dihydroprogesterone was comparable between the groups same as in our study. However, they observed no differences in the urinary concentration of allopregnanolone while we observed higher concentration of this steroid and its sulfate in plasma of ASD girls compared to CTRL. Higher concentration of allopregnanolone was found in saliva of <4 years and around 7–8 years old ASD girls compared to matched controls [27]. Our study showed that also other metabolites of progesterone, except 17-hydroxyprogesterone, like 16α-hydroxyprogesterone, 17, 20α-dihydroxy-4-pregnene-3-one and its conjugate were higher in ASD girls. Further metabolic steps include the formation of 5α/β-pregnane derivates which the most of them were higher or tended to by higher in ASD girls compared to CTRL. 5α/β reduced derivates of progesterone are neuroprotective anti-inflammatory steroids positively affecting myelination of Schwann cells so regulate neurogenesis, and, e.g. influence mood, memory, etc. [47]. Progestogens detected in amniotic fluid have already been associated with ASD development [5]. Whether they exert pathological or the opposite protective effect remain unclear.

Interestingly, allopregnanolone deficit during pregnancy has been related to postpartum depression [48]. Placentally derived allopregnanolone, a positive allosteric modulator of GABA_A_ receptors, is known to be essential during fetal neurodevelopment. Its lack e.g. due to preterm birth may cause neurological impairments and disorders including ASD [49]. This was also confirmed by different ASD animal studies. An animal model of akr1c14 KO mice causing insufficiency of placental allopregnanolone induced an autism-like behavior in male offspring. Moreover, investigating the cerebellum of these mice as well as cerebellum of pre-term birth male individuals showed some similarities e.g./like higher concentration of pdgfra- and cspg4-mRNA suggesting the proliferation of oligodendrocyte progenitor cells demonstrating that defect in allopregnanolone might contribute to abnormal brain development [50]. Allopregnanolone administration was capable to reverse ASD like behavior in in animal model of autism induced by administration of SKF representing an inhibitor of 5α-reductase I and II, which are indispensable for allopregnanolone biosynthesis. Interestingly, ASD-like behavior using this model was successfully induced only in male mice [51]. Allopregnanolone is also a negative modulator of glycine receptors and its sulfate is a positive modulator of NMDAR receptors [37]. Taken together, our data showing a trend to higher concentration of GABAergic steroids in ASD group and the aforementioned neuroprotective effects of these compounds indicate a contra-regulatory mechanism mostly mitigating the adverse effects of ASD. The higher levels of allopregnanolone sulfate (along with elevated pregnenolone sulfate) may also contribute to enhanced cognitive performance in ASD patients [37].

Even when the concentration of free DHEA was comparable between ASD and CTRL, DHEA sulfate, androstenediol, androstenediol sulfate and androstenedione were higher or tended to be higher in ASD, respectively, which can be understood as a higher level of TST precursors in the Δ^5^ pathway in ASD group. Androstenedione studied in plasma of adult females showed differences between women having Asperger Syndrome or high functioning autism and controls. On the other hand, no differences in DHEA, total and free TST were found between those groups [52]. Other above-mentioned complex studies found higher concentration of salivary DHEAS while no differences in salivary androstenediol and androstenedione in pre-pubertal girls [27]. However, significantly higher concentration of androstenediol and a trend toward higher DHEA was observed in urine of pre-pubertal girls [18].

Looking at the ratio of testosterone to epitestosterone in samples, Gasser et al. [53] showed lower ratio of these two steroids in urine of pubertal girls pointing to the higher concentration of epitestosterone in autistic girls.

Regarding the estrogens, estrone sulfate was observed to be lower in ASD group in comparison with CTRL group. Prenatally studied inactive estrone, along with the active female sex hormones estradiol and progesterone, have been found to be contributing factors to ASD risk in the future generation [5]. For CYP19A1 aromatase, reduced activity in the ASD group might be expected because a lower ratio of estrone sulfate to androstenedione was observed. Other study revealed no association of estrogens (estrone, estradiol, and estriol, and estetrol) and androgens (TST androstenedione) or their ratio detected in an umbilical cord blood with autistic-like traits measured in the future generation by Autism Quotient questionnaire [54]. Adrenal androgens play a role as immunomodulating and immunoprotective steroids. DHEA and its sulfate stimulate the secretion of some cytokines (e.g. IL-2) while suppress the other (e.g. TNF- α) and this activity is modulated and affected by other hormones. DHEA together with androstenediol are precursor of estrogens and both are also active on/work via estrogen receptors [55, 56]. Dysregulation of immune system and related inflammation is widely associated with ASD [57]. Taking together, many above mentioned studies point to relation of steroids with ASD allowing us to bring the pieces of puzzle together. It seems that cooperative effect of steroids across the whole pathway could stand behind the etiopathogenesis of such a complex disorder as ASD is. At this point it is only speculative to say which of them play protective or harmful role.

In this study, the alternative backdoor pathway of androgen production toward the synthesis of dihydrotestosterone evidently takes a place in both analyzed groups. Steroid precursors involved in this pathway like androsterone and its sulfate and epiandrosterone sulfate were higher in ASD group. Also, steroids like 17-hydroxyallopregnanolone and androstenedione were non-significantly higher in ASD pointing to the augmented activity of this pathway. On the other hand, the individual PPRs reflecting CYP17A1 in the lyase step are ambiguous because children at this age lack the activity of adrenal *zona reticularis*, so these conversions may be performed in different tissues i.e. kidney [58].

As mentioned above, sulfated steroids were in general higher in ASD group. C21 Δ^5^ steroids, so pregnenolone and its isomers, then C19 Δ^5^ steroids as well as progestins and androstanes were found to be more sulfated in ASD group in comparison with CTRL group. These results point to augmented steroid sulfotransferase (SULT2A1) activity or inadequate steroid sulfatase (STS) activity in ASD children. Besides adrenal *zona reticularis* and *zona fasciculata*, SULT enzymes are also expressed in the intestine, liver as well in the kidneys and lungs but to a lesser extent. SULT2A1 dominantly responsible for the sulfation of androgens and pregnenolone in children before adrenarche is most abundant in adrenal *zona fasciculata*. Moreover, some types of SULT enzymes take place also in the brain resulting in possible neuromodulation [59].

Present study was aimed to investigate steroids a narrow, age restricted group of prepubertal female individuals with ASD. Despite observing the very valuable results, we are aware of several limitations accompanying this study. Firstly, the sample size is small, which limits the statistical power and significance of the results obtained. Probably, due to this reason many non-significant differences leading to trends toward lower/higher concentration of steroids in ASD group in comparison with CTRL group were observed. Lower number of diagnosed girls unable us to perform analyses on bigger sample size.

Unfortunately, we were not capable to detect several main steroid intermediates like progesterone, DHT, TST, aldosterone or estradiol via GC-MS/MS due to their undetectable levels presented in little children recruited into study. This method is demanding in terms of the amount of sample required for sample analysis, but allows the simultaneous detection of dozens of steroids. However, the limitations of the biological material did not allow us to analyze the mentioned steroids by other methods. We decided to analyze a wide range of steroids in steroidogenic pathways instead of choosing the main metabolites. This approach offered us the opportunity to see individual steps of steroidogenesis at the expense of evaluation of the main products. Considering only specific hormones/main intermediates may not allow a mechanistic explanation of a complex disorder such as ASD. Therefore multiple detailed monitoring of e.g. hormonal cascade is needed. Also, these hormones do not act independently and their action is controlled or influenced by many other factors [60, 61]. Moreover, it is very difficult to say whether these impacts are even traceable prenatally. Regarding the access to a child itself, it is much more available to follow or look for the postnatal trace left by the prenatal hormonal impact.

## Conclusion

To conclude, lower activity of CYP11B1 but higher concentration of corticosterone was observed in ASD prepubertal girls compared to age-matched neurotypical CTRL group. The Δ^5^ steroids affecting neuronal activity through modulation of various ionotropic receptors appear to be higher in the ASD group. Regarding enzyme activity, our results suggest higher SULT/lower STS activity and lower 17-hydroxylase activity in girls with ASD compared to CTRL girls. In summary, the steroid metabolic pathway in autistic girls before adrenarche shows differences in the concentration of many steroids throughout the steroidogenic pathway, as has been reported in previous studies focusing on boys with ASD. Outcomes of all of these studies could be considered from wider point of view e.g. based on their effect via appropriate receptors. Also, these results also reveal possible actions of steroidogenic enzymes that influence the activity of steroid hormones and neuroactive steroids. Looking for these differences in other age groups would be beneficial for the future biomarkers or therapeutic strategies.

## Supporting information

S1 File (PDF)
