# Supplementary material for: Steroidogenic pathway in girls diagnosed with autism spectrum disorders
Source: PLoS One. 2024 Dec 5;19(12):e0312933. doi: 10.1371/journal.pone.0312933 (PMC11620458; doi:10.1371/journal.pone.0312933)
Supplement: S1 File — (PDF) [file pone.0312933.s001.pdf]

Sample	IDcislo	IDACVA	Status	Anor_LLR	Vek	Preg	PregC	Preg20a	Preg20aC	Preg17	Preg17C	Preg16a	Preg16aC	DHEA
1	1	KOZO12D	A	14	3,67	0,34975	23,8832	0,44328	100,163	2,40461	3,70303	0,04271	13,2072	1,01313
3	2	BAX23	A	14	3,84	1,0096	40,2432	0,79747	192,629	1,84747	4,0199	0,03109	22,2899	0,4605
5	3	BAX42	A	14	3,85	0,71732	11,4814	0,6547	61,9735	1,7077	2,15573	0,06289	54,1186	0,40799
7	4	HAZU14D	A	14	4,16	0,49599	62,4246	1,07725	371,04	0,73894	1,77458	0,00296	13,5064	0,05027
9	5	JARO13D	A	14	4,75	1,79883	68,6385	1,44889	271,667	9,20262	10,1489	0,10822	0,07744	9,1023
11	6	PONA13D	A	14	4,78	1,35347	47,9371	1,23565	140,319	7,7805	6,92486	0,09789	22,0328	0,92187
13	7	KRKA14D	A	14	4,95	0,74248	17,2213	1,06603	115,644	2,26613	2,35154	0,04688	28,7221	0,70572
15	8	NAX5	A	14	5,92	2,28015	52,1189	1,87955	175,443	6,78061	6,55134	0,09631	3,48976	1,57529
17	9	BAX30	A	14	4,62	1,15281	39,9909	0,76877	212,384	6,40734	8,6161	0,06931	3,97275	0,84965
19	10	BÍLU12D	A	14	6,19	1,56265	64,6586	1,50436	290,258	6,88504	8,57655	0,13373	26,7999	2,35549
21	11	ZLNI12D	A	14	5,82	3,45033	55,7808	1,33616	162,316	10,1158	7,03503	0,19948	139,712	0,80098
23	12	BAX37	A	14	6,88	0,76333	17,0319	0,56385	71,4129	3,50017	2,95549	0,03719	9,34081	0,71706
25	13	TRDO16D	A	14	3,1	0,56491	39,6449	0,86736	259,703	1,72162	3,46803	0,11808	25,5199	0,34452
27	14	SODO12D	A	14	6,82	0,44704	48,1004	0,83591	267,967	2,07549	5,74029	0,04636	38,9383	0,72536
29	15	VEKR15D	A	14	3,76	0,67747	118,124	1,99385	882,616	3,41201	5,78516	0,05287	73,0444	0,5409
31	16	FLEL15D	A	14	4,41	0,86712	51,1809	1,05206	359,676	1,23897	3,39063	0,13791	30,1465	0,31779
2	1	K1	C	-14	3	1,8688	33,648	1,80523	210,301	1,28759	2,09426	0,09182	11,5759	0,41794
4	2	K2	C	-14	3	2,02171	44,6766	0,75094	56,1964	2,46641	4,42166	0,00352	1,59876	0,70621
6	3	K3	C	-14	3	0,63709	23,118	1,04673	262,218	0,5291	1,08372	0,04063	41,2815	0,24623
8	4	K4	C	-14	3	0,69887	21,7208	0,81365	265,684	0,46311	0,86636	0,01218	32,6481	2,12631
10	5	K5	C	-14	3	0,90132	27,2697	1,2072	103,682	1,65551	2,73725	0,04797	33,5863	0,58449
12	6	K6	C	-14	4	0,75268	23,2054	0,71387	180,569	0,52874	1,2976	0,01762	89,6301	1,87056
14	7	K7	C	-14	4	0,70801	26,2068	0,67044	193,471	0,46383	1,3305	0,01625	59,8474	0,17714
16	8	K8	C	-14	4	2,08537	34,1377	0,97192	101,96	1,1519	2,02322	0,00665	95,4872	0,24542
18	9	K9	C	-14	4	0,97723	28,2788	0,40059	126,704	1,8798	3,61337	0,02972	55,0443	0,56424
20	10	K10	C	-14	4	2,14589	62,0048	1,62698	191,734	12,949	10,4427	0,14849	71,0355	1,4218
22	11	K11	C	-14	4	1,49339	173,045	2,08463	2491,22	2,86679	6,04342	0,08434	70,9353	1,04339
24	12	K12	C	-14	5	2,02112	27,8757	1,07162	205,738	1,54558	2,49868	0,01411	35,5374	0,59344
26	13	K13	C	-14	5	5,71325	67,2544	3,22312	305,725	7,05981	5,99384	0,13149	72,102	2,79693
28	14	K14	C	-14	6	1,50606	25,7875	0,95409	101,584	1,85584	2,86401	0,05032	23,4256	0,31032
30	15	K15	C	-14	6	1,30593	10,7371	0,62631	37,9035	2,57027	2,27227	0,06723	42,9563	0,84722
32	16	K16	C	-14	6	1,75129	27,0607	1,29813	117,495	1,72934	2,90374	0,0651	25,5373	0,55993

DHEAC	DHEA7b	Adiol	AdiolC	AT16a	AT16aC	Prog20a	Prog20aC	Prog17	Prog16a	Prog17a20a	Prog17a20aC	A2	TC	EpiTC
178,765	0,24241	0,03097	26,5987	0,05306	12,8484	0,02162	0,27567	0,20011	0,05173	0,04711	1,33013	0,27941	1,42476	0,22527
52,3885	0,18495	0,05129	5,42536	0,0401	17,3301	0,12471	0,35426	0,39528	0,12196	0,21941	1,21536	0,14519	1,10959	0,042
17,3553	0,2212	0,03346	2,08247	0,00869	3,34372	0,01225	0,30059	0,40839	0,17476	0,05871	0,01843	0,22564	1,2489	0,11195
89,8203	0,15646	0,84138	19,3697	0,05498	3,40022	0,06059	0,61476	0,10763	0,01239	0,09459	4,74025	0,05279	0,892	0,26515
203,374	0,38853	0,07953	2,56941	0,114	23,0856	0,04216	0,52151	0,39135	0,08294	0,16353	2,52563	0,37385	0,70566	0,18774
113,645	0,20007	0,17128	13,1864	0,03821	3,7057	0,04852	0,37866	1,09603	0,24814	0,11328	1,59381	0,48997	1,65071	0,09224
81,6058	0,1222	0,08419	23,5331	0,00927	4,03206	0,03933	0,22223	0,25958	0,10411	0,08523	1,43631	0,19229	0,01964	0,0266
134,871	0,36114	0,02127	10,0074	0,24125	52,1321	0,01522	0,57888	0,34146	0,09534	0,19063	1,84406	0,13321	6,04773	0,31328
102,899	0,22015	0,03849	27,8689	0,04256	14,5977	0,0173	0,47255	0,61336	0,17156	0,0307	2,20642	0,34331	0,34192	0,15331
564,989	0,18183	0,23695	7,45487	0,06892	45,7855	0,05226	0,52437	0,35949	0,06306	0,15379	2,42301	0,61708	6,88942	0,58954
96,4233	0,18697	0,013	14,8029	0,0911	9,08663	0,17672	0,73428	6,44276	3,42078	0,51947	3,66027	1,97916	0,60716	0,20309
78,5534	0,14671	0,03164	13,4716	0,03556	2,69063	0,01927	0,07357	0,88851	0,43634	0,07645	1,39795	0,47199	4,99165	0,03411
27,6874	0,1975	0,08893	4,40246	0,16439	3,14389	0,10793	0,19332	0,37911	0,19515	0,191	0,71495	0,12665	6,39206	0,02589
303,754	0,19056	0,0106	85,5076	0,01734	44,9785	0,01408	0,47721	0,20012	0,07567	0,15277	3,57156	0,30005	0,37988	0,21468
251,558	0,27378	0,05503	4,84449	0,07669	29,3413	0,06335	0,56791	0,37856	0,07626	0,25223	5,98756	0,17388	9,78975	0,47687
64,5254	0,198	0,11135	19,3256	0,10523	73,767	0,01838	0,06544	0,14069	0,04581	0,10359	3,42494	0,14218	0,03514	0,99227
21,846	0,2223	0,04381	3,86496	0,04287	4,55972	0,13308	0,52201	0,14106	0,09977	0,0541	1,08055	0,02907	2,00814	0,1175
19,4081	0,49536	0,20454	21,723	0,02036	4,43134	0,14726	0,71957	0,42049	0,33417	0,01883	0,09886	0,34824	10,3839	0,13502
39,1478	0,16001	0,1194	0,71817	0,02296	8,4609	0,02445	0,57117	0,20358	0,14934	0,1304	0,97817	0,02974	2,08905	0,05526
16,8399	0,12758	0,00262	2,3679	0,01788	3,37657	0,04377	0,48919	0,09989	0,104	0,04284	0,72068	0,01952	0,3125	0,00999
76,374	0,27049	0,08059	11,5854	0,00236	2,77017	0,01513	0,38739	0,12749	0,09885	0,09498	1,10367	0,04304	0,56556	0,17438
18,0276	0,14269	0,01179	5,19336	0,00185	2,76862	0,01775	0,26471	0,03828	0,01403	0,02327	1,14848	0,00895	1,02224	0,24497
19,3672	0,1756	0,00314	12,6247	0,00513	2,72904	0,01855	0,4505	0,03031	0,01719	0,05294	1,95136	0,007	7,6469	0,07465
4,0421	0,45666	0,00183	28,2021	0,06071	2,02432	0,00592	1,24033	0,26233	0,39555	0,06833	0,35753	0,06535	0,12801	0,09942
90,8129	0,15983	0,02914	17,9973	0,02293	19,0507	0,01639	0,33583	0,21442	0,06449	0,0692	1,58481	0,11037	0,00801	0,14631
176,604	0,22337	0,00178	27,633	0,2025	29,7463	0,03956	0,44131	2,37677	0,87851	0,37883	4,61818	1,14136	0,29688	1,32325
885,714	0,24746	0,04603	1031,28	0,41484	923,832	0,13291	2,24858	0,24529	0,07951	0,10321	48,0214	0,2794	1,71386	1,13989
193,529	0,21872	0,20152	70,5674	0,01889	11,6795	0,12611	0,51085	0,22669	0,12199	0,14194	0,98507	0,27199	2,85151	0,14233
508,429	0,5372	0,45073	59,701	0,01833	33,5435	0,11589	0,89643	1,88837	0,46013	0,81873	4,46692	0,69681	0,75864	0,71912
20,176	0,21633	0,00381	6,0534	0,02068	4,63563	0,05496	0,34877	0,22964	0,21408	0,06751	0,00673	0,18324	1,75788	0,15522
15,3054	0,17121	0,48791	2,2205	0,07836	3,3812	0,01411	0,18953	0,64349	0,43626	0,0697	0,23273	0,31513	4,57497	0,21168
24,9774	0,18668	0,01885	20,5044	0,04577	4,39924	0,07148	0,3779	0,33003	0,19545	0,07335	0,60936	0,20021	0,2391	0,075

E1C	P3a5a	P3a5aC	P3b5a	P3b5aC	P3a5bC	P3b5bC	P3a5a17C	P3a5b17C	P5a20a	P5a20aC	P3a5a20a	P3a5a20aC	P3b5a20a	P3b5a20aC
0,03205	0,02673	1,47657	0,05844	3,2639	3,66197	0,75767	1,11306	0,81646	0,0351	0,03963	0,14568	9,18357	0,06866	76,7508
0,37718	0,02237	2,8142	0,1083	5,06937	2,05233	0,87526	2,97283	1,61813	0,07254	0,05955	0,48472	12,6622	0,13982	105,173
0,20597	0,02862	1,3242	0,06493	1,95732	2,20945	0,53047	1,34414	1,49988	0,06252	0,1321	0,27889	17,3012	0,61503	43,4993
0,14061	0,07508	4,39469	0,06794	5,8757	6,24593	1,21416	1,51656	2,14325	0,07098	0,25414	0,13285	54,3959	0,43186	218,253
0,14108	0,11036	2,60631	0,18258	6,65395	4,52291	1,30269	2,02544	1,13615	0,09223	0,04554	0,46201	17,5931	0,49674	191,827
0,01913	0,04789	2,43947	0,16847	5,19079	3,07578	1,17204	1,86233	2,62014	0,08835	0,05183	0,84927	15,0242	0,56993	74,7576
0,05608	0,03062	0,96523	0,06426	2,19158	1,36054	0,43333	0,79237	0,69021	0,04925	0,11211	0,31769	7,98035	0,09185	86,0595
0,36172	0,05063	1,63991	0,27314	5,52001	6,50405	1,0349	1,45188	1,71297	0,10778	0,13338	0,0214	63,3197	0,60967	134,059
0,02599	0,03291	1,09971	0,08216	4,5277	2,20576	0,87838	1,69667	0,82532	0,02708	0,14102	0,08867	19,4648	0,24581	130,475
0,22552	0,05066	1,89332	0,14092	5,42891	4,55085	1,13538	1,67952	1,32547	0,09537	0,01163	0,44065	38,1619	0,42062	194,426
0,24559	0,07002	2,29757	0,35373	5,24082	2,38501	0,90894	4,2693	5,53928	0,10209	0,29737	0,47631	23,7655	0,89799	110,665
0,08059	0,00454	0,74462	0,0479	1,91558	0,93356	0,26214	0,40654	0,41185	0,01319	0,18012	0,29476	10,1758	0,122	50,5974
0,08282	0,07867	2,91106	0,11817	3,60604	2,70111	0,80137	1,94754	2,08577	0,14977	0,01368	0,06461	26,803	0,06621	169,28
0,02762	0,02665	2,29624	0,03857	4,4707	2,55752	0,95349	1,46518	1,45578	0,06315	0,21717	0,47559	24,1274	0,19363	186,605
0,04356	0,05518	2,57419	0,03229	8,38757	5,82062	1,35401	1,72156	3,07327	0,05311	0,0167	0,03411	40,4417	3,08862	580,726
0,2501	0,01011	0,62626	0,06944	4,4941	5,4941	1,23947	0,57927	1,91313	0,05111	0,05381	0,17846	5,93266	0,03561	255,048
0,11471	0,0024	1,56204	0,22542	4,1848	3,26968	1,04516	3,0045	3,11647	0,08044	0,00886	0,99287	23,2466	0,8971	133,468
0,34393	0,00673	0,80526	0,20518	4,16901	1,19315	0,56952	2,24194	1,651	0,03346	0,05315	0,00282	10,6515	0,17716	51,2997
0,10621	0,01425	1,22717	0,03706	3,08049	2,55003	0,93894	1,94595	2,8792	0,08622	0,15302	0,35242	32,8314	0,14233	150,703
0,02848	0,02708	1,12527	0,06093	2,86802	3,42992	0,37759	0,95565	1,50134	0,04662	0,28928	0,64176	15,7789	0,03205	112,431
0,20408	0,03556	1,19306	0,07024	3,83384	2,03997	1,0491	1,38075	0,96263	0,08289	0,1468	0,13874	4,7817	0,11784	74,5008
0,19109	0,0019	0,48741	0,07546	2,19926	1,34083	0,3477	0,98009	1,85305	0,0205	0,07938	0,05411	17,2076	0,3796	152,347
0,21492	0,01627	0,51031	0,05324	2,45879	1,58769	0,46696	1,11779	1,88028	0,02887	0,17761	0,03128	34,4113	0,13814	139,935
0,64689	0,01986	0,62681	0,11966	3,55262	0,83313	0,54619	0,92649	1,34466	0,00929	0,1181	0,23126	4,08533	0,2663	87,4295
0,01801	0,01087	1,49952	0,03806	2,90184	1,16009	0,52415	1,17406	1,34465	0,0287	0,29488	0,50004	14,9267	0,11474	78,2483
0,2381	0,04244	2,09045	0,21184	4,99515	4,73115	1,23139	2,79004	6,2166	0,04042	0,56313	0,36162	27,9027	0,45769	115,311
3,76141	0,07609	3,8569	0,17752	12,6871	7,4096	2,44741	1,30995	2,72425	0,14928	0,28545	0,67097	65,615	0,51169	1584,12
0,07669	0,01842	0,84189	0,1866	2,36766	2,17342	0,29057	0,56858	1,26698	0,05661	0,11272	0,1547	16,1216	0,02603	115,965
0,34333	0,10022	2,57632	0,64221	6,87511	4,78038	1,45664	3,14757	3,33727	0,25857	0,1807	0,13552	18,8926	2,20976	200,995
0,55028	0,03975	0,6212	0,1735	3,02196	1,66528	0,56314	0,86285	1,42465	0,09121	0,06258	0,34781	4,28381	0,85024	65,0725
0,05827	0,00453	0,30949	0,13004	1,29119	0,64436	0,15957	0,54074	0,67886	0,04834	0,06635	0,06327	2,22308	0,12757	32,1147
0,38042	0,02914	0,5943	0,16754	3,02014	1,92529	0,56766	1,33548	2,07408	0,08945	0,07202	0,2466	7,15398	1,1204	67,8575

P5b20a	P5b20aC	P3a5b20aC	P3b5b20aC	P3a5a17a20a	P3a5a17a20aC	P3b5a17a20a	P3b5a17a20aC	P3a5b17a20a	P3a5b17a20aC	A5a	A3a5a	A3a5aC	A3b5a	A3b5aC
0,01096	0,05406	1,78926	4,98727	0,01061	21,6251	0,07613	3,80036	0,22262	32,3332	0,04459	0,09924	66,9906	0,01922	20,7424
0,02247	0,04848	1,16755	6,72335	0,01909	15,534	0,01258	0,35427	0,1823	11,2727	0,02348	0,12585	27,0869	0,06864	6,94326
0,01229	0,0546	1,52533	2,85752	0,00235	92,5919	0,00486	0,26767	0,17591	22,9032	0,02343	0,08185	21,2142	0,01008	6,14708
0,01849	0,161	5,79012	8,19656	0,01255	656,683	0,00464	0,27539	0,85651	87,619	0,00316	0,01629	253,48	0,02192	49,9215
0,05037	0,03913	1,80147	18,1904	0,09003	45,1461	0,09952	0,95854	0,42979	21,2897	0,05395	0,03683	123,048	0,02796	33,5315
0,13391	0,07992	3,23366	21,0757	0,05155	37839,4	0,07489	0,61365	0,36367	8,14387	0,03112	0,08727	190,651	0,09023	40,2305
0,01116	0,04911	1,62237	5,18876	0,00518	48,7497	0,02077	2,31478	0,14033	31,0192	0,01474	0,06258	59,2331	0,02113	14,8833
0,09856	0,05446	6,83718	52,1365	0,12464	48,0151	0,06554	0,29935	0,96412	25,2998	0,03091	0,15274	21,5656	0,0162	10,3035
0,05429	0,03919	1,95256	18,8969	0,01268	6,99824	0,06677	4,12871	0,16694	16,1642	0,00853	0,08562	51,7288	0,00918	14,3929
0,00477	0,02102	0,20707	28,8884	0,03883	100,977	0,06056	0,98491	0,80446	36,5919	0,04758	0,1515	169,308	0,04075	51,7934
0,0194	0,02741	3,10356	14,8705	0,00931	41,7944	0,00621	1,17858	0,24821	115,904	0,05959	0,22167	150,831	0,0577	29,1272
0,01942	0,0801	2,05866	40,9402	0,00083	177,076	0,02434	0,75122	0,1241	5,23455	0,00996	0,06211	105,984	0,02297	24,5194
0,16777	0,08443	4,37314	35,0072	0,12417	120,498	0,11284	0,2244	0,47826	24,9636	0,03861	0,09663	38,9542	0,06097	8,68885
0,02701	0,11798	3,90768	14,2282	0,0123	63,6355	0,00512	0,17767	0,32223	38,623	0,02721	0,09491	115,883	0,0341	30,2882
0,01785	0,04413	2,75231	24,9535	0,04207	306,841	0,0802	9,47324	1,14159	67,3192	0,00987	0,02806	95,7101	0,00836	18,7761
0,04781	0,25654	6,24347	13,1254	0,00239	3228,39	0,0176	2,26451	0,48484	56,8349	0,00913	0,08832	5,7832	0,00251	3,10077
0,07482	0,04681	4,84104	41,8639	0,00073	7,55015	0,00625	0,0518	0,19649	14,8503	0,00342	0,06195	26,0206	0,1257	5,58318
0,05052	0,06934	0,97512	43,8945	3,8E-05	31,2165	0,00047	0,04087	0,08798	0,99811	0,0328	0,08955	13,6848	0,02018	4,32497
0,01485	0,03331	3,88219	28,6209	0,0178	1074,21	0,06539	2,96755	0,41719	23,8599	0,00211	0,03669	32,8544	0,00989	10,1223
0,01287	0,16965	1,82395	7,29437	0,00079	44,9365	0,01399	1,37063	0,12117	24,7478	0,00114	0,01309	20,2499	0,04313	5,7264
0,00689	0,03694	1,2408	6,86417	0,00491	15,896	0,05047	6,01878	0,11654	17,2307	0,02449	0,07966	50,9404	0,00407	14,946
0,01728	0,09965	1,99632	19,5994	0,00074	22,036	0,00081	0,11342	0,17568	20,7571	0,00268	0,01914	18,2142	0,03659	3,87439
0,02014	0,05253	4,50177	44,797	0,00014	107,66	0,00108	0,18213	0,15453	29,9167	0,00592	0,01174	21,1205	0,00877	4,05273
0,07957	0,13573	0,9435	3,2487	1,7E-05	25,5254	0,00049	0,12319	0,0204	8,63008	0,16202	0,00792	5,30515	0,00983	1,01705
0,01177	0,02705	0,94411	2,43619	0,0022	31,0037	0,00104	0,33058	0,08833	23,0011	0,00898	0,0149	36,3525	0,01291	12,4093
0,02014	0,50496	6,09021	9,57633	0,03844	28,0652	0,05898	1,66561	0,86688	60,8813	0,04125	0,20916	102,806	0,05821	21,4053
0,02564	0,12841	16,7547	11,7366	0,00804	6726,11	0,02244	15,5802	0,39435	247,707	0,02099	0,16971	173,648	0,01359	58,5475
0,01777	0,03373	2,18328	20,425	0,00653	427,774	0,00131	0,04399	0,51604	14,87	0,04387	0,01371	236,082	0,07605	33,654
0,02309	0,12428	2,88156	8,59193	0,16198	11,5406	0,13129	7,52082	1,54669	165,097	0,0595	0,32928	382,333	0,21929	114,778
0,03233	0,03113	1,33633	6,60763	0,00304	24,2821	0,03086	0,30758	0,14379	7,73441	0,03545	0,00705	9,20042	0,03984	2,87398
0,03851	0,02304	0,60073	0,4352	0,00088	16,6716	0,00997	0,17898	0,13742	5,24784	0,03019	0,04792	19,5634	0,06933	5,48525
0,02323	0,09408	2,15922	5,60377	0,00753	14,7024	0,04705	0,61553	0,22034	14,671	0,03312	0,08071	10,9848	0,0443	3,61083

A3a5b	A3a5bC	A3b5bC	A3a5a17bC	A3b5a17bC	A3a5b17bC	A3b5b17bC	F	B	A211b	A3a5a11b	A3a5a11bC	A3b5a11bC	A3a5b11b	A3a5b11bC
0,01941	2,04054	1,49925	0,98347	2,23004	0,09288	0,01431	205,532	2,97332	15,0443	0,57556	9,4283	2,79139	0,1542	33,7971
0,00814	0,88451	0,40279	0,35583	0,75064	0,14588	0,00987	297,239	6,35127	18,0165	2,20086	9,61012	1,10098	0,57703	12,7506
0,03335	1,24991	0,75122	0,41737	0,60101	0,12473	0,03183	215,491	6,77699	16,1419	1,09379	5,5475	0,5578	0,57855	24,7667
0,04199	3,40638	0,77635	3,32367	7,43831	0,13801	0,0072	166,939	0,81978	5,45127	0,76015	2,38218	0,4815	0,43435	86,6323
0,00646	1,51932	0,93181	1,41353	0,6712	0,10189	0,02357	199,332	3,77371	14,9921	1,07	4,45828	0,97104	0,15634	23,9304
0,05477	3,05046	1,42273	1,85807	3,51011	0,15348	0,01646	293,892	16,2393	24,0611	1,32365	5,18582	0,32691	0,75794	9,29243
0,01151	0,93217	0,42914	0,88891	2,84161	0,02095	0,01755	230,391	8,99971	16,5004	0,81705	3,47834	0,13777	0,38317	32,7045
0,07043	1,93577	0,61445	0,68783	0,75727	0,10638	0,01628	210,247	7,21956	23,6776	1,02765	13,5363	0,71118	0,61478	28,3477
0,01655	1,11134	1,86233	0,74346	3,67158	0,00535	0,02549	273,426	13,4537	21,0604	0,9858	7,20094	2,11057	0,35698	17,9605
0,0541	5,48747	3,01963	2,20057	0,64248	0,43862	0,27203	194,857	5,18612	15,6615	0,70498	8,96505	1,8605	0,44512	38,0859
0,08871	2,61203	0,66137	1,76096	2,48401	0,24618	0,12477	504,345	110,477	42,9078	2,39211	8,98797	0,57761	1,11215	112,498
0,01869	2,48081	3,2005	1,33256	3,62021	0,06278	0,00384	290,691	23,4171	26,1477	0,87564	5,36148	1,31806	0,74053	6,13649
0,11397	0,94586	0,40969	0,50736	1,21405	0,10194	0,02726	320,847	7,7839	21,2416	2,16081	3,94391	0,51253	1,7931	26,5978
0,03347	3,80672	0,8719	2,02244	4,532	0,16338	0,29753	211,515	7,15483	13,0533	0,89358	7,36193	0,25928	0,37201	40,0673
0,05686	2,29515	0,59579	1,55975	0,23555	0,12323	0,01493	140,065	3,20103	8,07115	1,61617	4,72217	1,54392	0,85108	67,3234
0,03456	0,98908	0,29274	0,25048	0,45484	0,07473	0,0131	120,34	2,48873	6,08617	0,34633	1,96129	0,2849	0,57137	57,5832
0,04277	1,00178	0,42934	0,27427	0,72191	0,11817	0,00749	256,812	4,11326	28,951	4,69988	9,00482	0,47475	2,36815	16,8328
0,038	0,49278	0,24095	0,39052	0,7568	0,31279	0,01954	367,379	14,0038	30,8635	1,73626	4,39127	0,15531	0,47034	1,37196
0,03034	2,34165	2,86104	0,49792	1,5668	0,27912	0,03965	266,15	6,32383	14,7921	1,35017	6,2928	2,89549	0,89355	25,4412
0,01805	0,73094	1,31036	0,2981	0,53645	0,00579	0,00765	136,268	2,42658	7,90752	1,93372	4,25837	1,00203	1,14076	26,2124
0,01673	1,85685	1,02806	0,74528	0,8294	0,04274	0,02915	119,181	2,43659	11,803	1,84466	3,73541	1,37351	0,99329	19,0542
0,00525	0,5439	0,21714	0,33693	0,7814	0,14762	0,00092	245,764	1,87843	16,4536	2,17477	3,20049	0,2206	1,12928	22,4453
0,00915	0,54919	0,21202	0,38009	0,89361	0,10185	0,00843	223,892	1,85393	13,6783	1,74778	3,16851	0,25127	0,75984	31,5242
0,00922	0,43441	0,29839	0,36942	0,12305	0,34116	0,10788	363,588	19,9286	23,1912	1,57454	2,64387	0,12003	0,92633	10,396
0,02345	1,11141	0,42021	0,55395	1,49243	0,02058	0,08591		1,60055	8,7914	1,95781	5,70537	0,16794	1,04405	24,4717
0,09329	7,00127	1,78189	1,96427	2,57932	0,22705	0,00722	416,64	26,7962	23,8725	2,37801	14,8618	3,63156	1,67291	61,0293
0,04414	17,2796	3,35134	5,55523	22,5626	0,73714	0,01899	136,921	2,77051	7,22129	0,57401	10,6877	1,6594	0,49875	228,598
0,06084	4,76587	3,10207	3,48159	7,9228	0,19422	0,02172	125,541	2,82953	25,3527	2,92175	7,38743	0,24711	1,68868	16,2486
0,08651	8,39659	7,74386	4,1809	8,02724	0,32286	0,06046	369,354	3,30653	37,7445	5,69426	14,0115	3,28073	1,60045	164,677
0,03698	0,67374	0,16714	0,22206	0,2415	0,26581	0,02714	213,514	5,83003	17,7327	1,95272	3,41832	0,25979	0,77252	8,91714
0,0471	1,15026	1,21812	0,24836	0,78021	0,14005	0,00891	409,825	22,4624	36,6444	3,58561	3,47562	1,62063	1,84317	6,22522
0,02986	0,82327	0,21602	0,27778	0,33988	0,31368	0,00837	216,525	5,54109	18,0153	2,15111	4,74576	0,40846	0,80475	16,193

Volume
950
750
770
870
440
900
860
390
700
900
890
900
900
900
940
900
550
350
930
1000
710
850
900
350
1000
1000
950
1000
400
800
740
860
